# Chinese herbal medicine for assisted reproduction technology: A protocol for a systematic review and meta-analysis

**DOI:** 10.1097/MD.0000000000022009

**Published:** 2020-09-11

**Authors:** Liangzhen Xie, Jian Li, Yan Li, Bingmei Wang, Chunyu Xie, Qing Xia, Zhigang Zhang, Ying Wang

**Affiliations:** a First Affiliated Hospital, Heilongjiang University of Chinese Medicine; b Harbin Medical University; c Department of Gynecology, the First Affiliated Hospital, Heilongjiang University of Chinese Medicine, Harbin, China.

**Keywords:** assisted reproductive technology, Chinese herbal medicine, protocol, systematic review

## Abstract

**Background:**

Human assisted reproductive technology (ART) has become an important part of infertility treatments throughout the world, including IVF, ICSI, embryo culture, and embryo cryopreservation. In China and East Asia, Chinese herbal medicine (CHM) has been used to treat various diseases and improves the success chance of live birth among infertile couples undergoing ART treatment. The aim of this study is to assess the effect and safety of Chinese herbal medicine among women undergoing ART.

**Methods:**

Cochrane Library, MEDLINE, EMBASE, CNKI, VIP, CBM and WANGFANG will be searched. All randomized controlled trials will be included if they recruited participants undergoing ART for assessing the effect and safety of Chinese herbal medicine. Primary outcomes will be live birth. Two authors will independently scan all the potential articles, extract the data and assess the risk of bias using Cochrane tool of risk of bias. Based on the guideline of Cochrane Collaboration, all analysis will be performed by RevMan 5.3 software. Dichotomous variables will be expressed as RR with 95% CIs and continuous variables will be reported as MD with 95% CIs. If possible, a fixed or random effects models will be conducted and the confidence of cumulative evidence will be assess using GRADE.

**Results:**

This study will be to assess the effect and safety of Chinese herbal medicine among women undergoing ART.

**Conclusions:**

This study will assess the effect and safety of Chinese herbal medicine among women undergoing ART and move forward to help inform clinical decisions.

## 1 Background

Human assisted reproductive technology (ART) has become an important part of infertility treatments since it emerged in the 1970s.^[1–3]^ The widespread application of assisted reproductive technology, such as in vitro fertilization (IVF), intracytoplasmic sperm injection (ICSI), embryo culture, and embryo cryopreservation, has delivered hope to countless 4 million infertile couples and the total number of live births attributable to ART have increased more than fivefold from 14,507 to 76,930 throughout the world.^[4]^ But the success rate of ART is not high at present, still hovering around 30% to 40%.^[5–8]^ The main reasons lead to low pregnancy rate among women undergoing ART including poor egg quality, decreased ovarian function, decreased endometrial receptivity and higher rate of threatened abortion caused by controlled ovarian hyperstimulation.

Chinese herbal medicine (CHM) has been used to treat various diseases in China and East Asia based on the diagnostic patterns of Chinese medicine (i.e., inspection, listening, smelling, inquiry and palpation), which is a 3000-year old holistic system. It typically consists of complex prescription composed of a combination of multiple components. Recently, Chinese herbal medicines that could improve the success chance of live birth among infertile couples undergoing ART treatment has been widely used all over the world^[9–12]^ Its mechanism may be to improve egg quality, ovarian function and endometrial receptivity, and avoid ovarian hyperstimulation during ART.

Up to present, one published systematic review has been conducted to summarize the evidence on the Chinese herbal medicine for the management of IVF-ET.^[13]^ However, there is no reliable evidence on the effectiveness of Chinese herbal medicine for ART because of few RCTs included in the analysis and the high risks of bias. Meanwhile, there are some published papers of CHM for ART. Therefore, it is of great importance to perform systematic reviews and meta-analyses of the randomized controlled trials (RCTs) on the effects of Chinese herbal medicine for ART. In this study, we will conduct a systematic review and meta-analysis of RCTs to evaluate the current evidence on the effects of Chinese herbal medicine on live birth rate among women undergoing ART, and move forward to help inform clinical decisions.

## 2 Methods

### 2.1 Objectives and registration

This review will be to assess and summarize the available evidence of Chinese herbal medicine on live birth among women undergoing ART. This review protocol is adhere to the Preferred Reporting Items for Systematic Reviews and Meta-Analyses Statement (PRISMA-P)^[14]^ and registered in the OSF platform (https://osf.io/registries) with a registration number 10.17605/OSF.IO/CQ4JR.

### 2.2 Eligibility criteria

#### 2.2.1 Type of study design

All randomized controlled trials involving Chinese herbal medicine for ART will be included in this systematic review regardless of publication status and language. Quasi-randomized trials (QRCTs) and non-randomized studies will be excluded.

#### 2.2.2 Types of participants

Adult women undergoing ART will be included regardless of their age, or race, educational and economic status.

#### 2.2.3 Types of interventions

All types of Chinese herbal medicines will be included with no limitations of the number of herbs, administration methods, dosage or duration of intervention. In included RCTs comparisons will be Chinese herbal medicine versus no treatment, placebo or other therapeutic agents before, during and after ART treatments.

#### 2.2.4 Types of outcomes

The primary outcome will be live birth which is defined as delivery of a live-born infant (≥20 weeks’ gestation). Secondary outcomes will include ongoing pregnancy, clinical pregnancy, multiple pregnancy, miscarriage, ovarian hyperstimulation syndrome and adverse events.

### 2.3 Information sources and search strategy

Seven electronic databases will be searched including Cochrane Library, MEDLINE, EMBASE, Chinese BioMedical Database (CBM), China National Knowledge Infrastructure (CNKI), Chinese VIP Information (VIP) and Wangfang Database regardless of publication status or language with the MeSH terms (“assisted reproduction technology” or “assisted reproductive treatment” or “in vitro fertilization” or “fertilization in vitro” or “intracytoplasmic sperm injection” or “oocytes” or “egg collection” or “embryo transfer” or “embryo implantation”) and (“Chinese herbal medicine” or “herbal” or “traditional Chinese medicine”).

### 2.4 Selection of studies and data extraction

**Figure 1:**
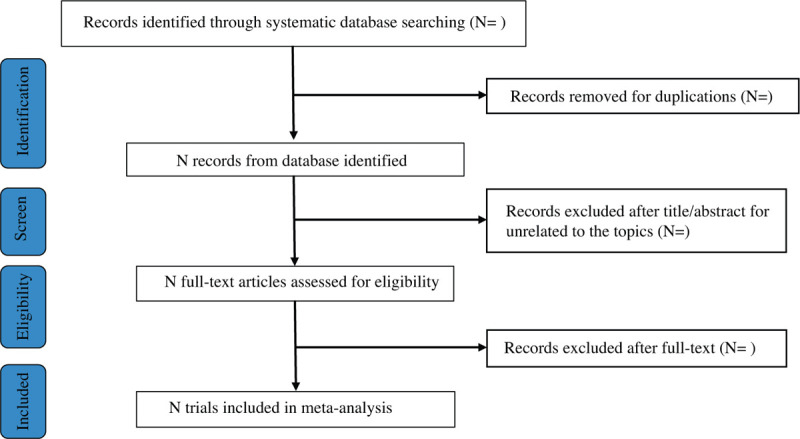
Flow chart of study selection.

All potentially relevant articles will be retrieved and organized in the Mendeley desktop. After an initial screening of titles and abstracts retrieved by the search, 2 reviewers (XLZ and LJ) will independently retrieve the full texts of all potentially eligible studies. Two reviewers (XLZ and LJ) will independently examine the full-text articles for compliance with the inclusion criteria. For the included studies, two reviewers (XLZ and LJ) will independently extract data and using a standard data extraction table designed according to Cochrane guidelines, including publication of year, author, participants, intervention, control, duration of intervention, outcomes and methodological characteristics. If there is any disagreement on the selection of articles and the process of data extraction, they will be discussed with the third author (LY). The study selection procedure will be shown in a PRISMA flow chart (Fig. 1).

### 2.5 Assessment of the risk of bias

Two reviewers (XLZ and LJ) will independently assess the risk of bias using the Cochrane tool of risk of bias (V.5.1.0). Six potential items will be assessed including random sequence generation (selection bias), allocation concealment (selection bias), blinding (performance bias and detection bias), incomplete outcome data (attrition bias), selective outcome reporting (reporting bias) and other bias. The judgments of evaluated domains will include high, low and unclear. Disagreements will be resolved by discussion by arbiter (WY).

### 2.6 Assessment of reporting biases

In view of the difficulty in detecting and correcting for publication bias and other reporting bias, we will minimize their potential impact by ensuring a comprehensive search for included studies and by being aware of duplicated data. Moreover, we will use funnel plots to explore the possibility of a small study effect, where there are sufficient studies. If asymmetry of funnel plots suggest possible small study effects, we will cautiously explain the results.^[15,16]^

### 2.7 Assessment of heterogeneity

According to Cochrane Handbook for Systematic Reviews of Interventions, we will use the I^2^ statistic to examine heterogeneity for quantifying inconsistency in all included studies. Where I^2^ value is greater than 50%, substantial heterogeneity will be indicated.

### 2.8 Data synthesis and statistical analysis

Based on the guideline developed by Cochrane Collaboration, we will perform statistical analysis using RevMan 5.3 software. We will express continuous variables as mean difference (MD) with 95% confidence intervals (CIs). For categorical variables, we will calculate risk ratios (RR) with 95% CIs. In this review, we will include all parallel-designed studies. For cross-over trials, we will include and analyze only the first treatment period data. For studies with multiple control groups, the unit of analysis will be used to each of all control groups. For insufficient or missing data, we will contact the authors by e-mail or phone as much as possible. All analysis will be performed based on intent-to-treat (ITT) principle. We will conduct a fixed-effect model when I^2^ < 50% or a random-effect model will be performed.

### 2.9 Subgroup analysis and sensitivity analysis

Subgroup analysis will be performed in order to explore the differences in the methodological quality, types of ART, race/ethnicity and types of herbal medicine. To assess the robustness of data analysis, sensitivity analysis will be conducted.

### 2.10 Confidence in cumulative evidence

In this study, the level of evidence on outcomes will be assessed using an approach based on the Grading of Recommendations Assessment, Development and Evaluation (GRADE).^[17]^ The quality of the body of evidence will be assessed based on five factors, including study limitations, effect consistency, imprecision, indirectness and publication bias. The assessments will be categorized as high, moderate, low and very low quality.

## Author contributions

**Conceptualization:** Liangzhen Xie, Jian Li, Yan Li, Ying Wang.

**Data curation:** Liangzhen Xie, Jian Li, Qing Xia.

**Formal analysis:** Liangzhen Xie, Qing Xia.

**Funding acquisition:** Liangzhen Xie, Jian Li, Bingmei Wang, Chunyu Xie, Ying Wang.

**Methodology:** Liangzhen Xie, Yan Li, Ying Wang.

**Project administration:** Jian Li.

**Software:** Liangzhen Xie, Qing Xia, Zhigang Zhang.

**Supervision:** Liangzhen Xie, Jian Li, Yan Li, Ying Wang.

**Visualization:** Liangzhen Xie, Jian Li, Yan Li, Bingmei Wang, Chunyu Xie, Ying Wang.

**Writing – original draft:** Liangzhen Xie, Jian Li, Yan Li, Qing Xia, Zhigang Zhang, Ying Wang.

**Writing – review & editing:** Liangzhen Xie, Jian Li, Yan Li, Bingmei Wang, Chunyu Xie, Qing Xia, Zhigang Zhang, Ying Wang.
